# The Overexpression of *Zea mays* Strigolactone Receptor Gene *D14* Enhances Drought Resistance in *Arabidopsis thaliana* L.

**DOI:** 10.3390/ijms25021327

**Published:** 2024-01-22

**Authors:** Chen Zhang, Fanhao Wang, Peng Jiao, Jiaqi Liu, Honglin Zhang, Siyan Liu, Shuyan Guan, Yiyong Ma

**Affiliations:** 1 College of Life Sciences, Jilin Agricultural University, Changchun 130118, China; zc17604665420@163.com (C.Z.); wangfanhaos@163.com (F.W.); 2 College of Agronomy, Jilin Agricultural University, Changchun 130118, China; m18404319202_1@163.com (P.J.); 17769351725@163.com (J.L.); 18743552271@163.com (H.Z.); 17769351725@126.com (S.L.); 3 Joint International Research Laboratory of Modern Agricultural Technology, Ministry of Education, Jilin Agricultural University, Changchun 130118, China

**Keywords:** *Zea mays*, strigolactone, *D14*, *Arabidopsis thaliana*

## Abstract

Strigolactones (SLs) represent a recently identified class of plant hormones that are crucial for plant tillering and mycorrhizal symbiosis. The *D14* gene, an essential receptor within the SLs signaling pathway, has been well-examined in crops, like rice (*Oryza sativa* L.) and *Arabidopsis* (*Arabidopsis thaliana* L.), yet the research on its influence in maize (*Zea mays* L.) remains scarce. This study successfully clones and establishes *Arabidopsis D14* gene overexpression lines (OE lines). When compared with the wild type (WT), the OE lines exhibited significantly longer primary roots during germination. By seven weeks of age, these lines showed reductions in plant height and tillering, alongside slight decreases in rosette and leaf sizes, coupled with early aging symptoms. Fluorescence-based quantitative assays indicated notable hormonal fluctuations in OE lines versus the WT, implying that *D14* overexpression disrupts plant hormonal homeostasis. The OE lines, exposed to cold, drought, and sodium chloride stressors during germination, displayed an especially pronounced resistance to drought. The drought resistance of OE lines, as evident from dehydration–rehydration assays, outmatched that of the WT lines. Additionally, under drought conditions, the OE lines accumulated less reactive oxygen species (ROS) as revealed by the assessment of the related physiological and biochemical parameters. Upon confronting the pathogens *Pseudomonas syringae* pv. *tomato* DC3000 (*Pst* DC3000), post-infection, fluorescence quantitative investigations showed a significant boost in the salicylic acid (SA)-related gene expression in OE lines compared to their WT counterparts. Overall, our findings designate the SL receptor *D14* as a key upregulator of drought tolerance and a regulator in the biotic stress response, thereby advancing our understanding of the maize SL signaling pathway by elucidating the function of the pivotal *D14* gene.

## 1. Introduction

Maize (*Zea mays* L.) is one of the pre-eminent cereal crops across the globe, with one third of the world’s population depending largely on this staple and its derivatives for food, leading the charge in the global grain output [1]. Maize’s development is susceptible to a multitude of detrimental factors that can precipitate significant yield losses, repercussions that can ripple through economies [2]. These adverse influences are predominantly abiotic, including factors like chilling injury, soil salinity, and drought, as well as biotic factors, such as various pathogens. The emergence of any such factor has the potential to inflict substantial damage on maize growth and production [3,4,5,6].

The strigolactone (SL), discovered at the dawn of the 21st century, emerged as a pivotal plant hormone initially identified for its germination-inducing effect on parasitic seeds, hence being considered for use as a germination stimulant [7]. Further research uncovered the role of SLs in modulating a wide array of physiological processes in plants, with extensive studies conducted on an assortment of species, such as sorghum (*Sorghum bicolor* (L.) Moench.), wheat (*Triticum aestivum* L.), rice, and *Arabidopsis* (*Arabidopsis thaliana* L.). It has been subsequently discovered that SLs have a far-reaching influence on plant growth, casting a regulatory effect on various growth factors, including the development of lateral roots and buds, leaf shape, the elongation of the main stem, and the processes of plant aging and development [8,9].

Coded by the *D14* gene, the D14 protein belongs to the α/β-hydrolase superfamily and exhibits both enzymatic and receptor functions [10]. Previous investigations unveiled three distinctive signaling entities within SLs, including the α/β-fold hydrolase At D14/D14/RMS3/DAD2, the leucine-rich repeat F-box protein MAX2/D3/RMS4, and the Clp protease family counterparts SMXL6/SMXL7/SMXL8/D53 [11]. In its dormant or ‘closed’ state, upon perceiving a signal, the D14 protein engages the activator of secondary signaling molecules, like MAX2, modifying its conformation to assemble into the Skp1–Cullin–F-box (SCF) complex. This interaction promotes the hydrolysis of SLs into intermediates, effectuating the degradation of the tertiary signaling molecules—Clp proteins. This critical cascade underpins regulatory mechanisms in plant architecture, notably in branching [12,13,14,15].

In nature, plants mainly resist biotic stress through three pathways: SA (salicylic acid), JA (jasmonic acid), and ET (ethylene) [16,17]. They cope with abiotic stress in the natural world through three pathways: ABA (abscisic acid), GA (gibberellin), and auxin [18]. The exogenous application of the SL analog GR24 has demonstrated an increase in drought resistance in winter wheat (*Triticum aestivum* L.) and *Pennisetum purpureum* Schum [19,20]. Conversely, in barley (*Hordeum vulgare* L.), variants bearing a missense mutation in the *HvD14* gene are found to be more vulnerable to drought-related damage [21]. Meanwhile, in *Arabidopsis*, the proteins SMXL6/7/8, acting as negative regulators for SLs, have been implicated in reduced drought tolerance [22]. Importantly, recent studies indicate that mutations in d14 and d17 exacerbate susceptibility to rice blast, suggesting the role for SLs in bolstering rice blast defenses by triggering an array of defense-related genes [23]. Additionally, the D14 protein has been reported to enhance drought tolerance in *Arabidopsis* [17]. Collectively, these findings imply that critical genes within the SL synthesis and signaling pathways are significant, not only in response to abiotic challenges, but also when influencing biotic stress reactions.

We developed overexpression lines (OE lines) in *Arabidopsis* harboring the maize *D14* gene. The results show that these OE lines have a notably longer primary root length when compared to wild-type lines (WT lines), along with decreased plant height in the tillering and germination stages, and a marginal reduction in the rosette phase and leaf area. Additionally, the OE lines displayed superior drought tolerance relative to the WT lines. Three days post-inoculation with the bacterium *Pseudomonas syringae* pv. tomato DC3000 (*Pst* DC3000), a notable elevation in the expression levels of crucial salicylic acid-related genes was observed, suggesting the potential role of the *D14* gene in plant defense mechanisms against pathogenic invasions. This investigation develops our insights into the drought stress responses and resistance processes mediated by the key SL receptor gene *D14*, as well as its potential involvement in hormonal interactions.

## 2. Results

### 2.1. Tissue-Specific Expression of the Maize D14 Gene

**Figure 1:**
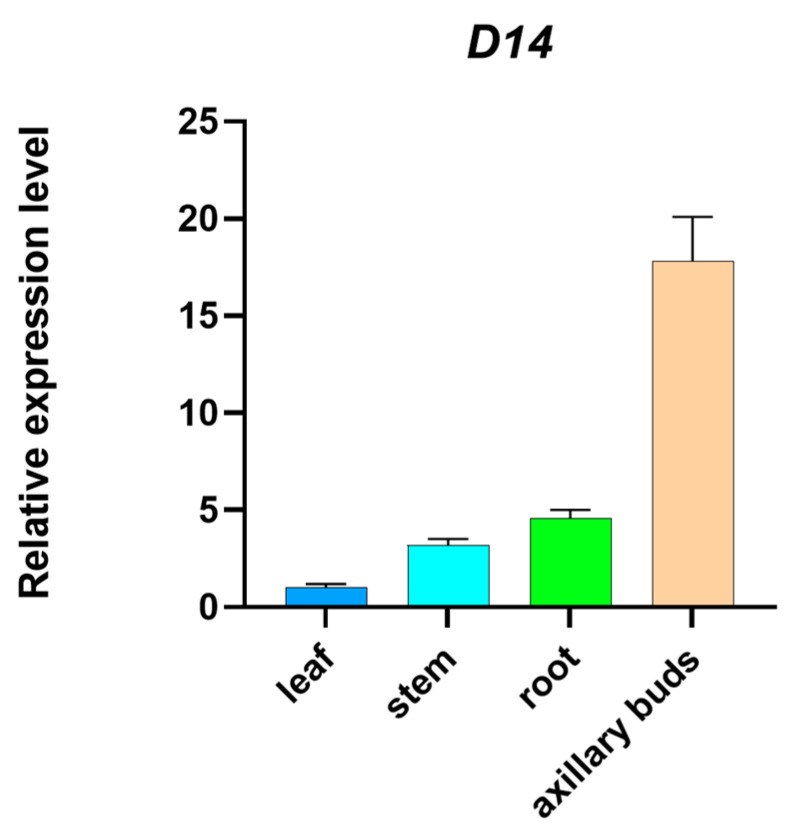
Changes in expression levels of maize *D14* gene in various parts of maize. The expression levels of leaf were normalized. The experiment was carried out in three replications and six maize plants were selected for each replication. The data are expressed as the mean of triplicate values, and the error represents the SD.

An examination of the relative expression variations of the maize *D14* gene was performed during the seedling phase. Tissue-specific expression patterns of the *D14* gene were investigated using maize B73-2 as the reference template. The findings demonstrate that, in the absence of any treatment, the expression levels of this gene are observed to be the highest in the axillary buds and lowest in the leaves (Figure 1).

### 2.2. Generation and Molecular Identification of Transgenic Arabidopsis

**Figure 2:**
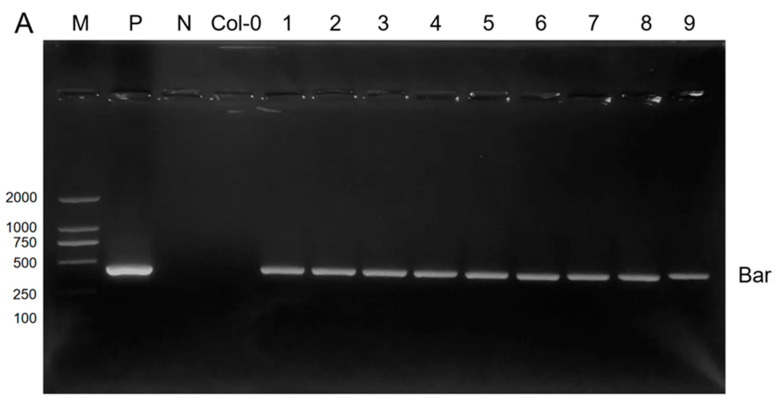

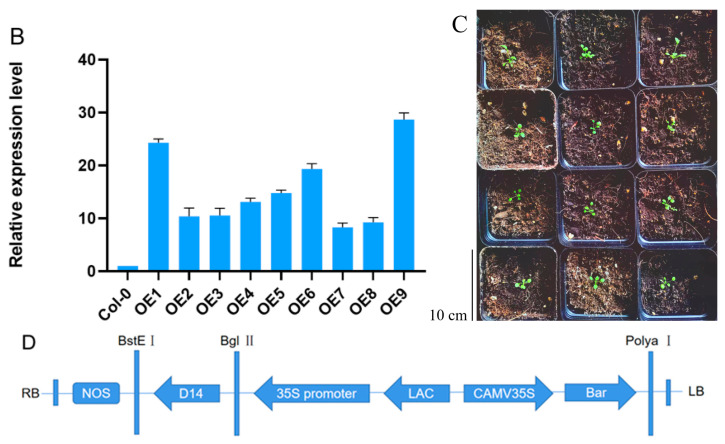
Production and molecular identification of transgenic *Arabidopsis*. (**A**) PCR detection of the *bar* gene in transgenic *Arabidopsis*, M: DNA marker DL 2000, P: pCAMBIA3301-D14 recombinant plasmid, N: negative control, a non-transgenic plant line (Col-0): non-transformed plant, 1–9: transformed pCAMBIA3301-D14-positive plant. (**B**) qRT-PCR validation of transgenic *Arabidopsis*. (**C**) Untransformed and transgenic plants. (**D**) Vector profile of pCAMBIA3301-D14. The expression levels of a non-transgenic plant line were normalized. The data are represented as three times the average of the duplicate values; error: SD.

We constructed the recombinant plasmid pCAMBIA3301-D14 and introduced it into the wild-type *Arabidopsis* Col-0 lines (a non-transgenic plant line), where the *D14* gene was overexpressed, resulting in nine transgenic lines (Figure 2A). The transgenic plants at the T3 generation were tested for fluorescence quantification (Figure 2B), and the three homozygous lines with the highest expression levels (OE1, OE6, and OE9) were selected for subsequent experiments (Figure 2C).

### 2.3. Phenotypic Analysis of Arabidopsis with D14 Gene Overexpression

**Figure 3:**
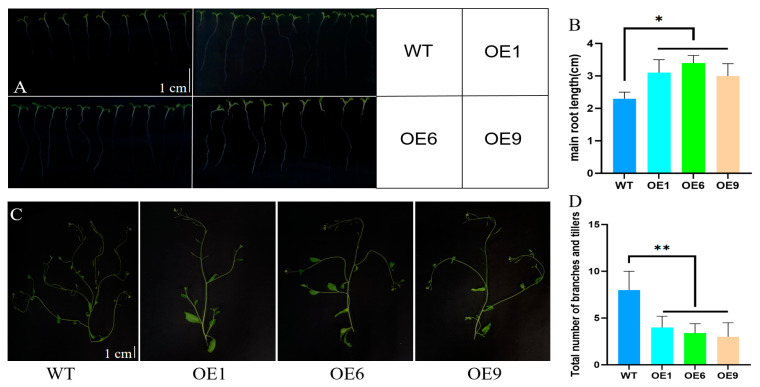

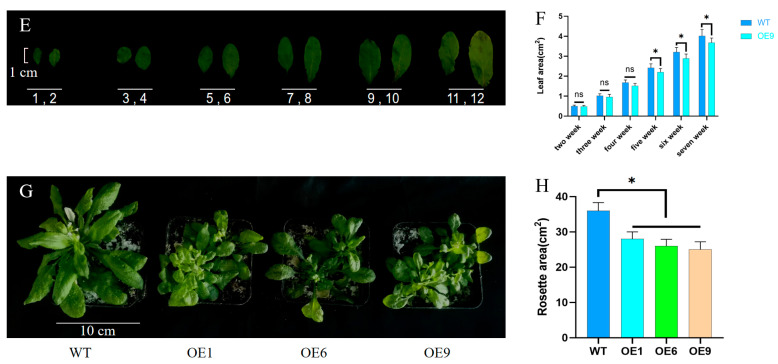
Phenotypic analyses of OE1, OE6, and OE9 lines and WT lines. (**A**,**B**) Primary root length of wild-type and overexpressor lines at 14 days post-germination. Experiments were repeated three times, with 30 *Arabidopsis* individuals measured per experiment, totaling 90 individuals across the three repeats. (**C**,**D**) Tiller and branch numbers of WT and OE lines. Experiments were repeated three times, with 30 *Arabidopsis* individuals measured per experiment, totaling 90 individuals across the three repeats. (**E**,**F**) Leaf area measurements for the WT and OE9 lines were cataloged weekly, spanning from the second to seventh weeks. For example, entries 1 and 2 correspond to the leaf areas of WT and OE9 lines when they are at two weeks of age, while 3 and 4 relate to the leaf areas at three weeks of age, proceeding in this manner. The study was conducted over three replicates, each involving measurements obtained from 30 individual *Arabidopsis* plants, culminating in an aggregate of 90 individuals measured. (**G**,**H**) Rosette leaf areas of wild-type and OE lines at 7 weeks of age. The greatest distance between two opposite leaves were measured, taking this value as the diameter to calculate the rosette leaf area based on the formula for the area of a circle. Experiments were repeated three times, with 30 *Arabidopsis* individuals measured per experiment, totaling 90 individuals across the three repeats. Data is represented as three times the average of the duplicate values, error said SD. Asterisks indicate significant differences: non-significant (ns), *p* < 0.05 (*) and *p* < 0.01 (**). WT: wild-type lines; OE1: overexpression line 1; OE6: overexpression line 6; OE9: overexpression line 9.

The overexpression lines OE1, OE6, and OE9 were germinated on ½ MS media to investigate the associated phenotypic variations during their germination. The data reveal unchanged germination rates (Figure S1a,b); however, the OE lines initiate germination marginally earlier than the wild-type (WT) lines (Figure S1c,d). The OE lines developed longer primary roots as opposed to the WT lines (Figure 3A,B). The OE lines’ heights were significantly reduced at four weeks old (Figure S1e,f). By the seventh week, the OE lines exhibited a reduction in the tiller count (Figure 3C,D), leaf area (Figure 3E,F), and rosette size (Figure 3G,H) in comparison with the WT lines. Furthermore, the OE lines underwent a quicker onset of senescence relative to the WT lines (Figure S1g,h).

### 2.4. Analysis of the Expression of Various Hormones in Arabidopsis with D14 Gene Overexpression

**Figure 4:**
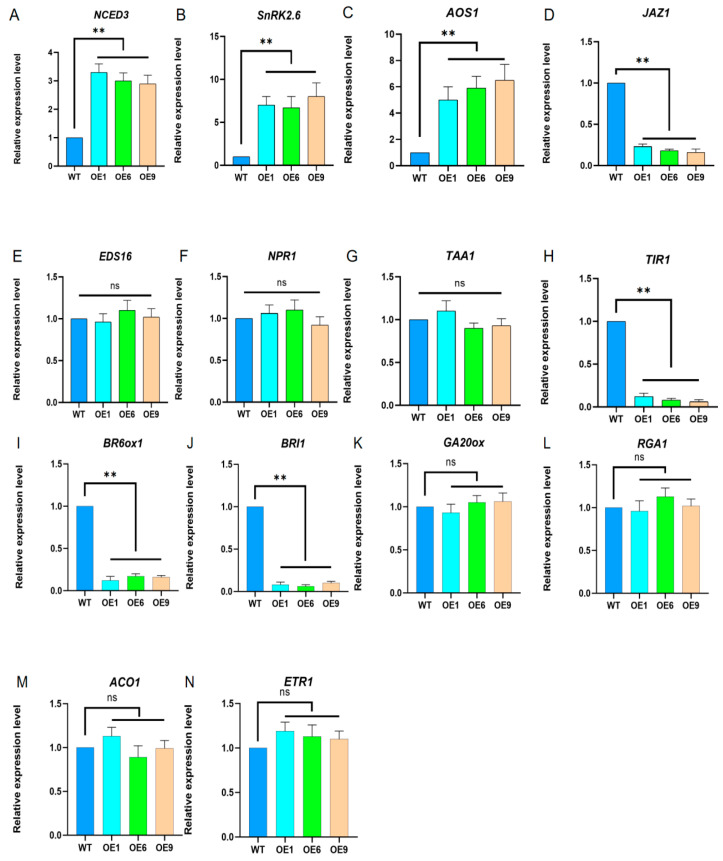
Analysis of hormone expression patterns in *Arabidopsis* with *D14* gene overexpression. (**A**,**B**) Abscisic acid (ABA) synthesis and detection of signaling pathway expression levels; (**C**,**D**) jasmonic acid (JA) synthesis and detection of signaling pathway expression levels; (**E**,**F**) salicylic acid (SA) synthesis and detection of signaling pathway expression levels; (**G**,**H**) auxin synthesis and detection of signaling pathway expression levels; (**I**,**J**) brassinosteroid (BR) synthesis and detection of signaling pathway expression levels; (**K**,**L**) gibberellin (GA) synthesis and detection of signaling pathway expression levels; (**M**,**N**) ethylene (ET) synthesis and detection of signaling pathway expression levels; the expression levels of WT lines were normalized. Using Student’s *t*-test, asterisks indicate significant differences: non-significant (ns) and *p* < 0.01 (**). Data are shown as mean ± SD from three independent experiments.

Considering the intricate interactions among plant hormones, we postulated that the D14 overexpression-mediated augmentation of strigolactone signaling could potentially influence the biosynthetic and signaling pathways of plant hormones in *Arabidopsis*. To test this theory, we chose seven plant hormones, namely, abscisic acid (ABA), jasmonic acid (JA), salicylic acid (SA), auxin, brassinosteroids (BRs), gibberellins (GAs), and ethylene (ET), for the investigation. For each hormone, a pivotal gene from its respective biosynthesis and signaling cascades was subjected to a fluorescence quantitative analysis. The findings indicate a significant upregulation in the expressions of ABA biosynthesis and signal transduction genes, *NCED3* and *SnRK2.6*, when compared with the WT lines (Figure 4A,B). Furthermore, there was a notable increase in the expression of the *AOS1* gene associated with JA biosynthesis, and a marked decrease in the expression of the JA signaling-related gene *JAZ1* (Figure 4C,D). The expression levels of the central genes *EDS16* and *NPR1* within the SA synthesis and signaling pathways remained unaltered (Figure 4E,F). In the auxin pathway, the crucial gene *TAA1* displayed no significant change in expression levels, while the expression of *TIR1*, a key player in auxin signaling, was significantly repressed (Figure 4G,H). Markedly lower expression levels were observed for *BR6ox1* and *BRI1*, vital genes in the BR biosynthesis and signaling pathways (Figure 4I,J). The GA-related genes *GA20ox* and *RGA1*, important for GA synthesis and signaling, showed no significant alterations in expression (Figure 4K,L). Finally, the expression levels of *ACO1* and *ETR1*, key players in the ET biosynthesis and signaling pathways, did not exhibit significant changes (Figure 4M,N).

### 2.5. Overexpression of Arabidopsis Improves Survival after Drought-Recovered Water

**Figure 5:**
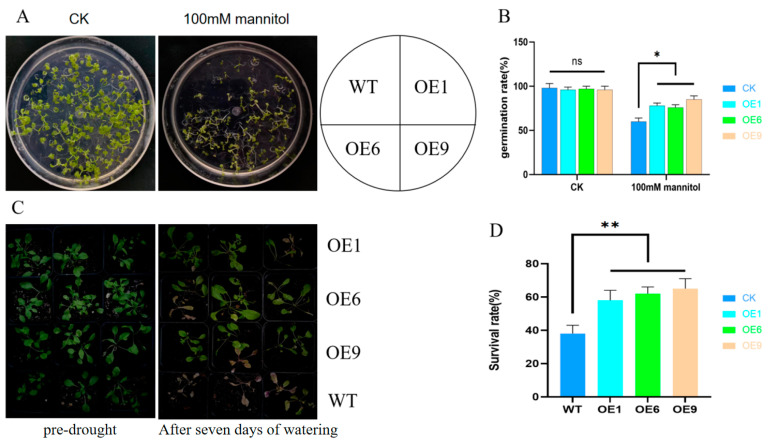
Overexpression of *Arabidopsis* lines showed some resistance to drought stress. (**A**,**B**) Drought stress was simulated using 100 mM of mannitol. Experiments were repeated three times, with 100 *Arabidopsis* seeds measured per experiment, totaling 300 seeds across the three repeats. Effective germination was defined as a root formation of ≥3 mm. The diameter of the petri dish is ten centimetres. (**C**,**D**) Re-watering experiments of 4-week-old *Arabidopsis* under drought conditions were repeated three times, with 30 plants used per experiment, totaling 90 plants across the three repeats. Using Student’s *t*-test, asterisks indicate significant differences: non-significant (ns), *p* < 0.05 (*), and *p* < 0.01 (**). Data are presented as mean ± SD from three independent experiments.

We evaluated the drought stress responses of OE1, OE6, OE9, and WT *Arabidopsis* lines at the germination stage and at four weeks post-germination. Our study revealed that the OE lines exhibited a markedly higher germination rate compared to the WT lines following a 14-day exposure to 100 mM of mannitol during germination (Figure 5A,B). To further assess *Arabidopsis*’ drought tolerance, we carried out a dehydration and subsequent rehydration trial on plants at four weeks of age, subjecting them to 15 days of drying followed by 7 days of re-watering and monitoring the phenotype and survival. The OE lines showed a significantly improved survival rate over the WT lines after the 7-day rehydration period (Figure 5C,D). To ascertain the OE lines’ resilience to salinity and cold, we introduced salt and cold stress at the germination stage. After a 14-day period under 100 mM of NaCl salt stress, there was no appreciable difference in the germination rates between the WT and OE lines (Figure S2a,b). Moreover, the germination rates after a 14-day cold stress period at 12 °C did not differ significantly between the WT and OE lines (Figure S2c,d).

### 2.6. Overexpression of D14 Gene Reduced ROS Accumulation under Drought Stress Conditions in Arabidopsis

**Figure 6:**
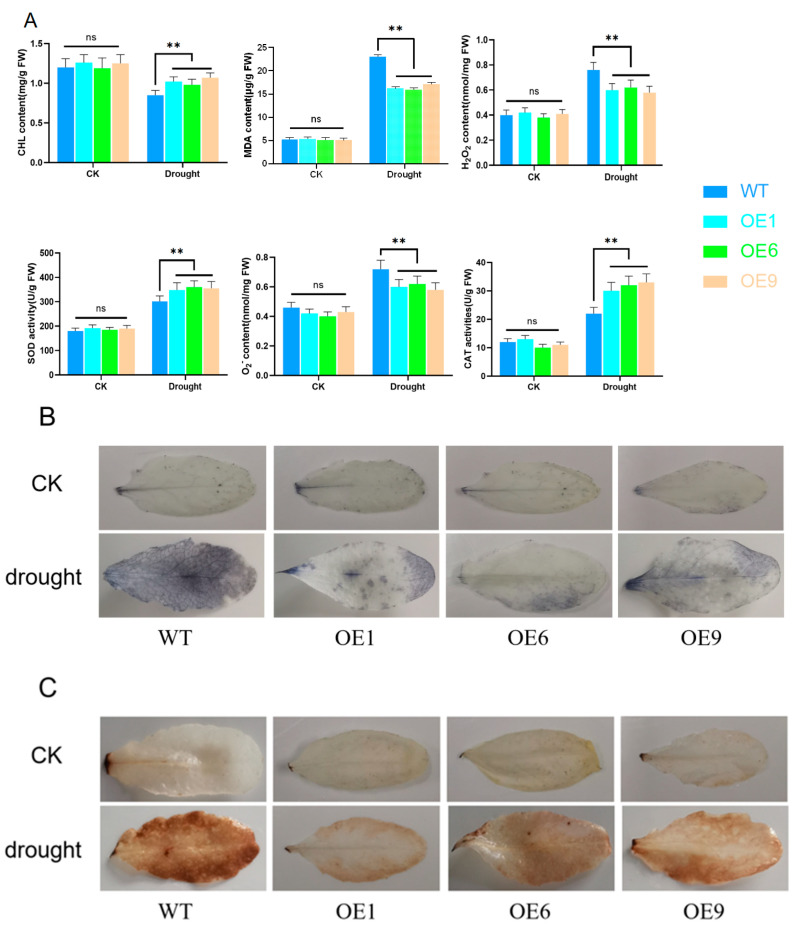
Overexpression of *D14* gene reduced reactive oxygen species (ROS) accumulation under drought stress conditions in *Arabidopsis*. (**A**) Determination of the contents of chlorophyll (CHL), malondialdehyde (MDA), hydrogen peroxide (H_2_O_2_), superoxide dismutase (SOD), superoxide anion (O_2_^−^), and catalase (CAT) in leaves; (**B**) NBT staining; (**C**) DAB staining. Using Student’s *t*-test, asterisks indicate significant differences: non-significant (ns) and *p* < 0.01 (**). Data are shown as mean ± SD from three independent experiments.

To assess the effects of *D14* on the biochemical and physiological attributes of *Arabidopsis*, we measured the chlorophyll (CHL) and malondialdehyde (MDA) levels in both WT and OE lines. To determine the capability of *D14* overexpression in diminishing reactive oxygen species (ROS) accumulation, we analyzed the concentrations of hydrogen peroxide (H_2_O_2_), superoxide dismutase (SOD), superoxide anion (O_2_^−^), and catalase (CAT) (Figure 6A). Furthermore, the levels of ROS were evaluated using nitroBlue tetrazolium chloride (NBT) staining and 3,3′-diaminobenzidine (DAB) staining (Figure 6B,C). In normal growth conditions, no substantial differences were detected in the CHL, MDA, H_2_O_2_, O_2_^−^, and CAT levels between the WT and OE lines. Nonetheless, when exposed to drought stress, the WT lines exhibited a pronounced reduction in CHL, SOD, and CAT contents, and a notable increase in MDA, H_2_O_2_, and O_2_^−^ levels compared to the OE lines.

### 2.7. Changes in Transcription Levels of Key Genes Related to Drought Resistance Pathways in Arabidopsis

**Figure 7:**
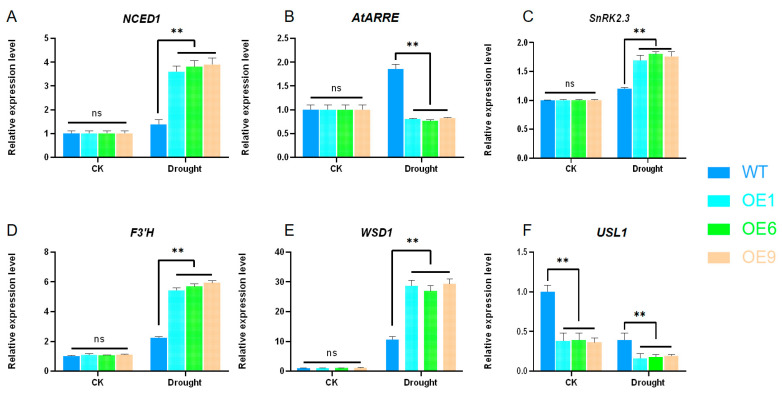
The expression levels of drought-related genes in 6 different pathways were analyzed in *Arabidopsis*. The expression levels of WT lines were normalized. (**A**) Changes in *NCED1* gene expression before and after drought stress in WT lines and OE lines. (**B**) Changes in *AtARRE* gene expression before and after drought stress in WT lines and OE lines. (**C**) Changes in *SnRK2.3* gene expression before and after drought stress in WT lines and OE lines. (**D**) Changes in *F3’H* gene expression before and after drought stress in WT lines and OE lines. (**E**) Changes in *WSD1* gene expression before and after drought stress in WT lines and OE lines. (**F**) Changes in *USL1* gene expression before and after drought stress in WT lines and OE lines. Using Student’s *t*-test, asterisks indicate significant differences: non-significant (ns) and *p* < 0.01 (**). Data are shown as mean ± SD from three independent experiments.

During our investigation, *Arabidopsis* specimens at four weeks of age were exposed to simulated drought conditions using 15% polyethylene glycol (PEG-6000). The findings from this exposure suggest that, relative to the WT lines, OE lines exhibit a pronounced upregulation of the *NCED1* gene (Figure 7A) after 8 h of aridity. Moreover, a substantial downregulation was observed in the *AtARRE* gene (Figure 7B), whereas the expressions of the *SnRK2.3* (Figure 7C), *F3′H* (Figure 7D), and *WSD1* (Figure 7E) genes were each significantly upregulated. Contrastingly, the *USL1* gene expression was notably diminished (Figure 7F).

## 3. Discussion

The hormone inhibition hypothesis suggests that strigolactones (SLs), commonly synthesized as secondary messengers in the roots, possess a function to repress the growth of axillary buds. In alignment with this hypothesis, our investigation revealed that the *D14* gene, responsible for encoding the principal receptor for SLs in maize (*Zea mays* L.), exhibited a predominant expression in the axillary buds (Figure 1) [24]. Moreover, previous reports have shown that rice (*Oryza sativa* L.) d14 mutants exhibit an increased tillering phenotype, with a notably higher endogenous SL content in the roots and root exudate compared to the wild type [25]. It was also demonstrated that rice mutants with defects in SL biosynthesis (d10-1, d17-1, and d27-1) could suppress the outgrowth of tiller buds under treatment with the synthetic SL analog GR24, unlike SL signaling mutants (d3-1 and d14-1), which failed to do so [26]. This ineffectiveness could be attributed to a lack of a sufficient SCF–ubiquitin complex assembly downstream of the D14 protein, precluding the efficient degradation of SLs and subsequent signaling activation. It is known that a single gene is unlikely to regulate a specific trait in plants, and the variability in traits may not be due to just one gene’s influence. Therefore, the specific mechanism by which SLs regulate the complex trait changes in *Arabidopsis* remains to be studied.

Our investigation revealed that the expression levels of several critical hormone-associated genes were markedly variable in the OE lines when contrasted with the WT lines (Figure 4). This suggests a heightened hormonal interplay in response to a heightened sensitivity to endogenous SLs, which in turn can have a direct or indirect impact on the biosynthesis and signaling pathways of other hormones. Specifically, genes *NCED3* and *SnRK2.6*, which are involved in the synthesis and signaling of ABA (abscisic acid), show considerably higher expression levels in the OE lines versus the WT lines. Since ABA facilitates seed dormancy and germination, this can partially account for the apparent earlier germination of OE seeds compared to WT seeds. Additionally, the *D10* gene’s expression level was distinctly decreased in the OE lines, whereas the expression levels of the signaling genes *D53* and *D3* were considerably elevated (Figure S3). This suggests the overexpression of the *D14* gene can instigate a compensatory response involving the *D53* gene, thereby causing an upregulation of *D53*, which in turn suppresses the expression of the *D10* gene, reducing SL synthesis and helping to sustain a relative hormonal equilibrium. Notably, in the OE lines, the genes integral to BR biosynthesis and signaling, such as *BR6ox1* and *BRI1*, are prominently reduced, an aspect also involved in seedling emergence and development. This can help explain the diminutive rosette conglomerate and leaf area relative to the WT lines, though it fails to elucidate the enhanced root elongation observed during the germination phase (Figure 3). The differential hormone expression profiles between the germination phase and at the four-week stage suggest underlying mechanisms warranting further elucidation.

Recent studies suggest that SLs can be governed by a long-distance feedback loop, primarily through diminishing the transport capacity of auxin, sequentially inhibiting SL synthesis [24,25]. Furthermore, in *Arabidopsis* SL-signaling mutants max1 and d27, a substantial increase in the expression levels of the *PIN* genes—those coding for auxin transport proteins—implies that SLs potentially regulate the polar transport of auxin by adjusting the expression of *PIN1*, consequently influencing plant architecture [26,27,28]. *SLR1,* considered a repressor of GA signaling, appears to be capable of degrading the bulk of GA-responsive elements. The absence of SLs in both the roots and root exudates of Slr1 mutants points to GA signaling as a negative regulator of endogenous SL synthesis [29]. Additionally, in rice treated with the SL analog GR24, there was a notable surge in the expression of *OsCKX9*, the gene encoding cytokinin oxidase. Yet, in the SL signaling mutant, d53, of rice, GR24 treatment did not alter the *OsCKX9* expression, indicating that cytokinin synthesis could be suppressed by SLs, whereas auxin could promote it. These observations imply that SLs are promoted by auxin, curbed by GA, and, alongside auxin, can concurrently inhibit cytokinin synthesis to modulate plant branching. The specific role of SLs in maize, compared to other plant species, remains to be further experimentally deciphered [30].

Plants known, to date, mainly employ three major pathways, ABA (abscisic acid), GAs (gibberellins), and auxin, to withstand abiotic stressors in nature, and changes in the expression levels of some key genes can affect plant resistance to abiotic stressors [18,31,32,33,34]. For example, in maize (*Zea mays* L.), lines overexpressing the ABA receptor genes *ZmPYL8*, *ZmPYL9*, and *ZmPYL12* show improved drought tolerance [35]. In studies on maize and tomatoes (*Solanum lycopersicum* L.), it was found that diminished endogenous levels of GAs enhanced the plant’s ability to withstand drought [36]. In rice (*Oryza sativa* L.), the increased expression of the *OsPIN9*, a member of the auxin efflux transporter PIN-FORMED (PIN) family, diminished the plant’s cold tolerance [37]. The RBOH-ROS-auxin signal transduction pathway was observed to help rice roots avoid heavy metal and salt-induced stress [38]. Our experiments confirmed that overexpressing the *D14* gene in *Arabidopsis* markedly strengthened its drought endurance (Figure 5), yet it did not seem to bolster its defense against salt and cold stresses (Figure S2). Nevertheless, SLs have been reported to also enhance certain plants’ tolerance to heat and cold stresses [39,40]. This difference in function can be related in some way to the heterologous expression of genes or differences between species.

While the intricacies of how various hormones mutually influence each other in the face of stress are not fully understood, it is a frequent observation that hormones modulate reactive oxygen species (ROS)-linked signaling pathways and peroxidase levels during drought episodes [41]. Hydrogen peroxide (H_2_O_2_) and superoxide radicals (O_2_^−^) are pervasive during periods of plant stress; plants usually generate greater quantities of these substances when confronted with biotic or abiotic stressors, initiating cellular programmed death and strengthening the plant’s stress resilience. These ROS levels can be indicative of the intensity of stress experienced by the plant [42]. The enzymes superoxide dismutase (SOD) and catalase (CAT) play crucial roles in scavenging ROS, and their levels of activity are often synonymous with a plant’s tolerance to adverse conditions [43,44]. Plants under duress produce substantial ROS, which leads to lipid peroxidation [45], and the levels of malondialdehyde (MDA), as lipid peroxidation’s end product, can also act as a measure of plant stress damage [46]. Drought stress prompts a decline in the chlorophyll content, which constrains photosynthesis. NBT staining indicated that OE lines maintained lower ROS accumulation under drought conditions when compared with WT lines. Furthermore, OE lines exhibited significantly reduced concentrations of H_2_O_2_, O_2_^−^, and MDA, and heightened activities of SOD and CAT relative to WT lines under drought stress conditions. These findings suggest that overexpressing the *D14* gene potentially lessens the damage to *Arabidopsis* caused by stress by boosting the activity of antioxidant enzymes and promoting the clearance of ROS (Figure 6).

To explore the expression patterns of genes linked to drought response and to delve into the mechanism of *D14*, several drought-related genes were chosen for an expression level analysis (Figure 7). *NCED1* is identified as a pivotal rate-limiting gene within the ABA biosynthesis pathway [47,48]. The ABA-associated RING-type E3 ubiquitin ligase in *Arabidopsis* (*AtARRE*) acts as a negative regulator of ABA accumulation [49]. The *SnRK2.3* gene responds to ABA signaling and abiotic stresses alike [50,51]. A key player in anthocyanin accumulation is represented by the *F3′H* gene [52].The *WSD1* gene is instrumental in the synthesis of cuticular waxes [53]. The RING-box protein-coding gene, *USL1*, is essential for ABA-mediated plant regulation and leaf aging [54,55]. These data imply the maize *D14* gene functions as a modulator in *Arabidopsis* during droughts, exerting a beneficial influence on drought response. Previous studies have shown that *Arabidopsis* mutants in SL biosynthesis, namely, max3 and max4, and the signaling mutant max2, are more vulnerable to drought stress [19]. In grapevines (*Vitis vinifera* L.), exogenously applied GR24 enhances drought tolerance, characterized by increased ABA levels, with stomatal closure regulated by ABA contributing to this effect and thus easing the impacts of drought [56,57,58]. Plants also respond to abiotic stresses by elevating ABA and decreasing GA levels [59]. The MYB96 MYB transcription factor in *Arabidopsis* integrates auxin and ABA signaling to modulate drought stress responses [60]. Raised internal ABA concentrations prompt stomatal closure during droughts as a stress response mechanism [60,61]. Genes, such as *F3′H* and *WSD1*, associated with anthocyanin synthesis and cuticle formation, respectively, increase their expression levels in response to droughts [54,55]. The ubiquitin-conjugating E2 enzyme subfamily member encoding gene, *USL1*, is pivotal in ABA-regulated plant processes and leaf senescence. Studies revealed that the expression of senescence-related genes in usl1 mutant mature leaves was heavily ABA-dependent, causing a marked increase in the ABA concentration and consequent leaf aging [62]. It can be inferred that the overexpression of *D14* under drought conditions can enhance anthocyanin production via an unidentified pathway and contribute to the reduction in water loss by upping the cuticular wax creation to lower the transpiration rate. Nonetheless, the precise mechanisms are yet to be elucidated.

Our study discovered a significant rise in salicylic acid gene expression post-*Pst* DC3000 infection, hinting at the potential role of the *D14* gene in the pathways mediating disease resistance (Figure S4). Importantly, the expression levels of pivotal genes in SA biosynthesis and signaling pathways in uninfected *Arabidopsis* plants did not vary considerably (Figure 4). This observation indicates that SLs likely instigate defense responses against pathogenic assaults following inoculation and potentiate SA gene expression via a hormonal interplay subsequent to infection, consistent with the previous research [63]. The function of SLs as an augmenting factor for plant disease resistance has been established across a range of species. For instance, the rice SL biosynthetic mutant d17 and the signaling-defective mutant d14 exhibited increased vulnerability to rice bacterial blight [23], while in tomatoes (*Solanum lycopersicum* L.), the SL deficit mutant ccd8 is more susceptible to the leaf pathogens *Botrytis cinerea* (Pers.) and *Alternaria alternata* (Fr.) [64]. The *CCD7* and *CCD8* genes are pivotal in the biosynthetic pathway of strigolactones [65,66]. In island cotton (*Gossypium barbadense* L.), the knockout of these genes results in diminished disease resistance. Conversely, in upland cotton (*Gossypium hirsutum* L.), the overexpression of the *GbCCD8b* gene correlates with an increase in disease resistance [67]. In the medium supplemented with GR24, many plant root pathogens showed growth inhibition, including *Fusarium solani* f. sp. *Mango*, *Sclerotinia sclerotiorum* (Lib.), *Colletotrichum acutatum* (J.H. Simmonds.), and *Botrytis cinerea* (Pers.) [68]. Collectively, these findings underscore the positive regulatory influence of SLs on plant disease resistance, with the exact pathways and mechanisms awaiting further discovery.

## 4. Materials and Methods

### 4.1. The Growth Conditions of Zea mays

Maize B-73 seeds were sown at a 2:1 ratio in a mixture of peat and perlite, respectively. The plants were cultivated to the three-leaf stage under a long photoperiod (16 h of light, 8 h of darkness), with daytime temperatures of 26 °C, nighttime temperatures of 20 °C, a relative humidity of 65%, and a 16 h/8 h day/night light cycle. RNA extraction and quantitative fluorescence analysis were conducted. During the growth of *Arabidopsis* transgenic plants, we used 50 watt fluorescent lamps as the light source. These lamps were of the LED (light-emitting diode) type, providing a spectrum similar to natural sunlight. The spectral range extended from 400 to 700 nm, covering the blue (450–495 nm) and red (620–750 nm) radiation regions required for plant photosynthesis. The fixtures were installed approximately 30 cm above the plant apex, providing a uniform illumination with a photon flux density of 1500 micromoles per second per square meter (μmol/s/m^2^), ensuring adequate light exposure for the plants to achieve optimal growth and development.

### 4.2. Production and Phenotypic Analyses of Transgenic Plants

The *D14* gene from maize was successfully cloned into the high-expression vector, pCAMBIA-3301, via Bgl II and BstE II restriction sites. This recombinant pCAMBIA-3301-D14 vector was transformed into the *Agrobacterium* GV3101, which was subsequently used to inoculate Col-0 through the floral dip method [69]. The selection of seeds occurred on ½ MS solid medium with kanamycin, leading to the identification of positive lines in the T3 generation. This study proceeded to select three lines exhibiting high expression levels of the *D14* gene, with the Columbia wild type (Col-0) serving as the comparative control. The growing conditions were controlled at a daytime temperature of 24 °C and a nighttime temperature of 20 °C, maintaining the relative humidity at 65%, with a light/dark cycle of 16 h light/8 h dark. The plants were cultivated in German K brand 876 peat soil. For the root length examination, the *Arabidopsis* seeds underwent a sterilization process using 75% ethanol for one minute and 1% sodium hypochlorite for ten minutes, followed by thorough rinsing with sterile water 3–5 times. These seeds were then evenly distributed for vertical growth on ½ MS medium. Germination assessments were conducted using similar sowing procedures on ½ MS medium. To evaluate the expression variations of pivotal hormone-associated genes, RNA extractions and quantitative fluorescent analyses were performed on four-week-old *Arabidopsis*. At the seven-week mark, photographic documentation along with assessments of tiller numbers, plant heights, and rosette areas were conducted.

### 4.3. Arabidopsis Abiotic Stress Treatments

For the evaluation of drought and salt stress tolerance in transgenic *Arabidopsis*, we utilized ½ MS media comprising 0 and 100 mM of mannitol, as well as 0 and 100 mM of NaCl. The WT and OE lines were uniformly distributed across these media and monitored, with photographs documenting germination and root development at intervals of the 3rd, 7th, and 14th days. When assessing the cold stress tolerance, we incubated the ½ MS media seeded with *Arabidopsis* at both 24 °C and 12 °C for a 14-day period, during which documentation via photography and data notations were conducted. During the drought recovery trial, the OE and WT lines of *Arabidopsis* were co-placed in a tray to which we added 1L of water, this ensured ample and uniform water absorption by both lines of *Arabidopsis*. Following a 24-h period for soil saturation, the leaves from the OE and WT lines were harvested to ascertain their weights. Concurrently, separate sets of leaves from both the OE and WT lines were immersed in distilled water for 24 h, achieving saturation before they were weighed (the initial leaf weights were virtually identical for the two measurements). A negligible difference in the weight comparison before and after saturation implied that the *Arabidopsis* in the tray was in a state of complete saturation. We marked this as day 0 (Figure 5C (pre-drought)) and initiated a 15 day drought period. After 15 days, we re-watered the tray with 1L of water, maintained it for 7 days, took photographs, and recorded the survival rates of the OE and WT *Arabidopsis* lines (Figure 5C (after 7 days of watering)). Each of these experiments was conducted in triplicate.

### 4.4. Culture, Infection, and Detection of Plant Pathogens

In our research, we utilized *Pst* DC3000 as the pathogenic strain for infecting the *Arabidopsis* plants. To initiate the activation, bacterial suspensions preserved at −80 °C were cultured in King B medium supplemented with 20 µg/mL of rifampicin at 28 °C for 24 h. To ensure sustained high pathogenicity, active bacterial colonies from the previous step were subsequently cultured for an additional 24 h in fresh KB medium using the same process.

The bacteria were eluted from the pathogen-containing medium with a 20 mL sterile 10 mM MgCl_2_ solution and then diluted to an optical density (OD) of 0.05 using sterile 10 mM MgCl_2_. Following the dilution, 200 µL/L of surfactant Silwet L-77 was added to complete the bacterial suspension mix. This suspension was evenly sprayed over the *Arabidopsis* leaves until droplets began dripping from the foliage. Afterward, the leaves were covered with a clear protective dome. The plants were then enclosed using transparent protective covers to ensure a consistently high humidity environment for a period of three days. Following this interval, the above-ground sections of the plants were harvested for an analysis through fluorometric quantification.

### 4.5. NBT (Nitroblue Tetrazolium Chloride) Staining

To prepare the NBT staining solution, dissolve 50 mg of NBT powder into 100 mL of Tris-HCl buffer (pH 7.4). Subsequently, submerge the processed *Arabidopsis* leaves in the NBT solution and allow it to stand for 2 h at room temperature, shielded from light. Following the incubation period, carefully extract the leaves with tweezers and place them into 95% ethanol. Proceed with the decolorizing process in an 85 °C water bath until all traces of green have been removed from the leaves. Once decolorized, retrieve the leaves for photographic recording.

### 4.6. DAB (3,3′-Diaminobenzidine) Staining

To create the DAB staining solution, combine 30 mg of DAB powder with 50 mL of distilled deionized water (ddH_2_O) until a concentration of 0.5 mg/mL is achieved. The prepared *Arabidopsis* leaves should then be submerged in this DAB solution and left to incubate at room temperature overnight, protected from the light. Following the incubation, extract the leaves and immerse them in 95% ethanol, decolorizing them in a water bath maintained at 85 °C.

### 4.7. Determination of Physiological and Biochemical Activities

For the quantification of the chlorophyll content, an Arabidopsis leaf tissue weighing 0.1 g was homogenized in a mortar with an 80% acetone solution until becoming a uniform slurry and subsequently incubated at 4 °C overnight. The resulting supernatant was then extracted for absorbance measurements at 665 and 649 nm wavelengths, utilizing the UV2400 UV/Visible spectrophotometer (Soptopo, Shanghai, China) [70]. MDA was detected using the detection kit [71]. The contents of H_2_O_2_ and O_2_^−^ were determined according to the acetone method [72]. A reaction mixture was prepared consisting of 50 mM of potassium dihydrogen phosphate buffer, 2 mM of Na^2-^EDTA, 0.1 mol/L of H_2_O_2_, and 0.2 mL of the enzyme extract for the analysis of catalase (CAT) activity. Concurrently, the variation in absorbance was recorded at a wavelength of 240 nm. A suspension was formulated containing 50 mM of potassium dihydrogen phosphate buffer, 120 mM of Met, 0.1 mM of EDTA, 700 μM of NBT, 60 μM of riboflavin, and 0.2 mL of enzyme extract, which was then used for the assessment of superoxide dismutase (SOD) activity [73].

### 4.8. qRT-PCR

In this study, the trizol method was used to extract the total plant RNA. The first cDNA strand was generated using the M-MLV reverse transcription kit (Takara, Kusatsu, Japan). The system and program used for qRT-PCR were Light Cycler^®^ 96 (Roche, Switzerland), SYBR green, and 40 cycles of 95 °C for 30 s, 95 °C for 10 s, and 60 °C for 30 s. The maize reference gene was *ZmActin1* and the *Arabidopsis* reference gene was *AtActin2*. The 2^−∆∆CT^ method was used for the data analysis. Each sample was repeated three times.

### 4.9. Data Analysis

All the results in this study were repeated more than three times, and GraphPad Prism 9 was used for the statistical analysis and plotting. Statistically significant differences in the model identifiers: *p* < 0.05 (*) and *p* < 0.01 (**).

## 5. Conclusions

Upon thorough investigation, it was found that *Arabidopsis* lines engineered to overexpress the maize strigolactone (SL) receptor gene, *D14*, not only presented substantial reductions in morphological traits, such as the plant height, number of tillers, and leaf area, but also an expedited physiological cycle, characterized by higher germination rates and an earlier onset of senescence, compared to wild-type lines. Additionally, these D14-overexpressing lines demonstrated a noticeably strengthened resistance to drought conditions when faced with trials involving cold, salt, and drought stresses. Pathogen challenges prompted a clear upregulation of essential salicylic acid genes in these plants. The results substantiate the role of the *D14* gene in modulating growth and development in *Arabidopsis* and imply its potential significance in the plant’s stress response arsenal. The growth and developmental alterations noted could relate to SLs’ influence on plant physiological and developmental pathways, while the observed improvements in drought resilience strongly hinted at SLs’ prospective role in helping plants cope with unfavorable conditions. Future studies are warranted to further examine *D14*′s function within maize itself, as well as how SLs’ signaling finely tunes plant physiological responses, with a particular focus on stress resistance. The results achieved from this research can offer novel theoretical insights into breeding resilient crop varieties and inform strategies to bolster crop robustness against environmental stresses.

## Data Availability

The data that were used are confidential.

## Supplementary Materials

The following supporting information can be downloaded at: https://www.mdpi.com/article/10.3390/ijms25021327/s1.
