# Supplementary material for: The Overexpression of Zea mays Strigolactone Receptor Gene D14 Enhances Drought Resistance in Arabidopsis thaliana L
Source: Int J Mol Sci. 2024 Jan 22;25(2):1327. doi: 10.3390/ijms25021327 (PMC10816222; doi:10.3390/ijms25021327)
Supplement: Supplementary file 1 [file ijms-25-01327-s001.zip › ijms-2743963-supplementary.pdf]

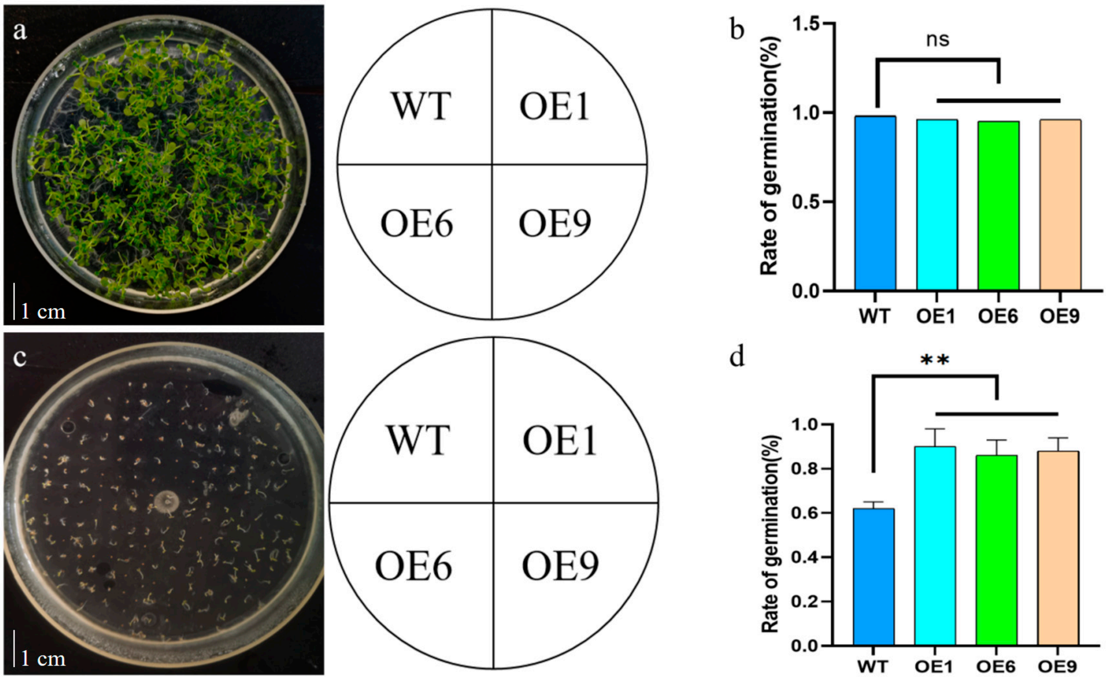

a
1 cm
WT
OE1
OE6
OE9
b
ns
Rate of germination(%)
1.5
1.0
0.5
0.0
WT
OE1
OE6
OE9
c
1 cm
WT
OE1
OE6
OE9
d
**
Rate of germination(%)
1.0
0.8
0.6
0.4
0.2
0.0
WT
OE1
OE6
OE9

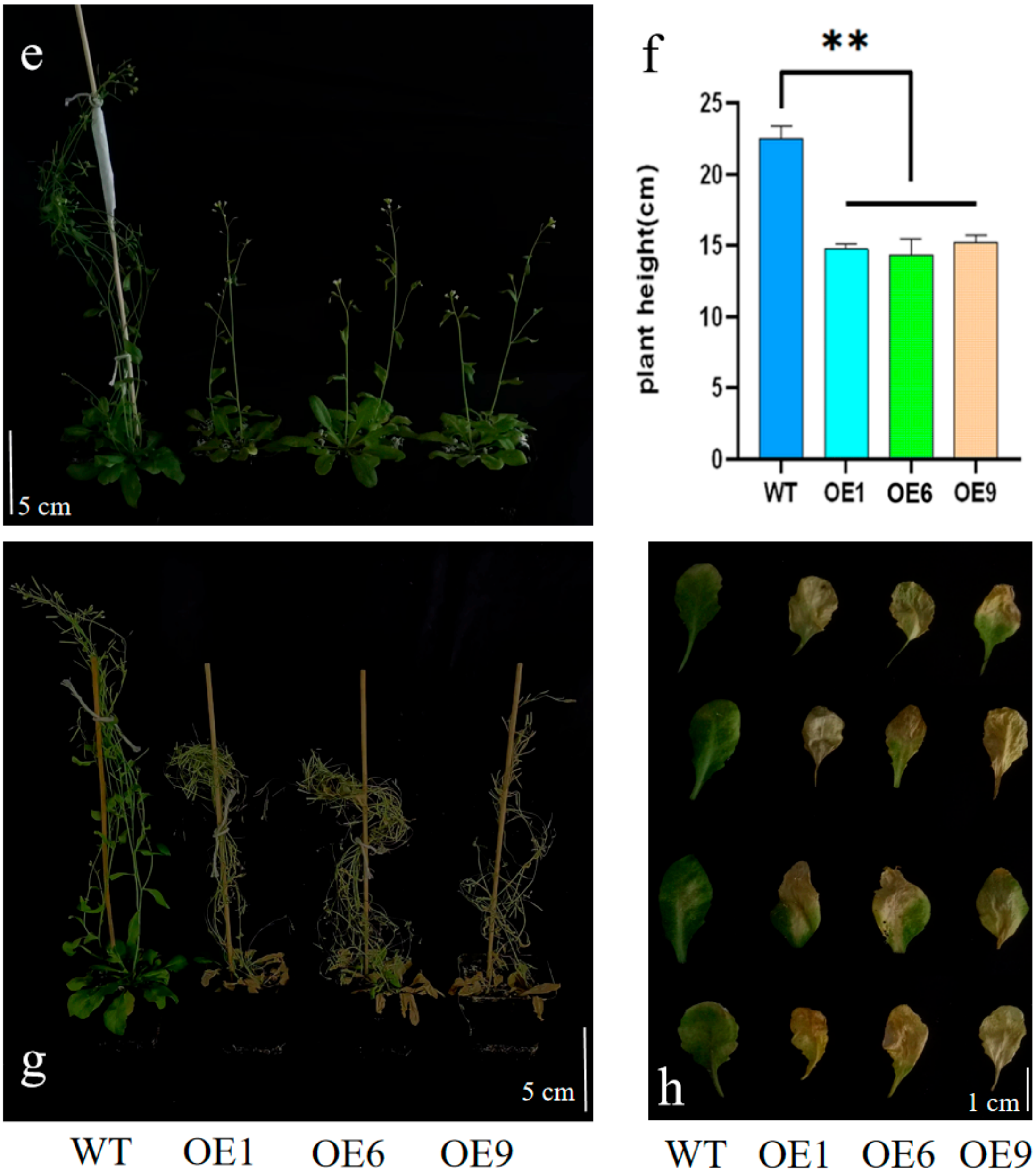

Figure S1. Phenotypic analysis of OE1,OE6, and OE9 lines and WT lines. (a, b) Germination rates of WT and OE lines at 14 days. The experiment was replicated three times, three petri dishes were sown each time, and the germination rate was calculated as the average of the three replications; (c, d) Germination rate of WT and OE lines at 3 days. Effective germination was defined as the formation of roots of 3 mm or more in length. The experiment was replicated three times and three petri dishes were sown each time, germination rate was calculated as the average of three replications; (e, f) Height of WT and OE lines at four weeks of age. The experiment was repeated three times, nine plants were selected for measurement each time, and the height was calculated as the average of three replications; (g, h) Aging analysis at eight weeks of age. Leaves were selected from the fifth leaf of the rosette; Data is represented as three times the average of the duplicate values, error said SD. Asterisks indicate significant differences: non significant (ns) and $p < 0.01$ (**).

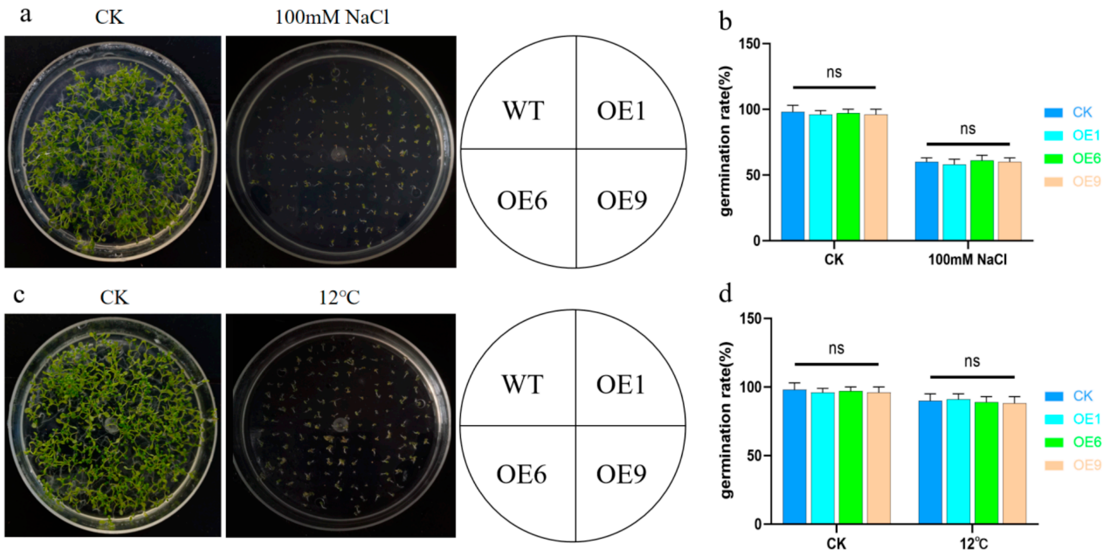

Figure S2. *Arabidopsis* overexpression lines did not show significantly enhanced tolerance to cold stress and salt stress. (a, b) Salt stress was simulated with 100 mM NaCl. The experiment was replicated three times, with two petri dishes sown for each experiment and 50 to 100 *Arabidopsis* seeds per dish. Effective germination was defined as the formation of 3 mm or longer roots. Germination was calculated as the average of three replications. (c, d) Simulation of cold stress at 12° C; in the germination stress experiment, the experiment was replicated three times, with three Petri dishes (one Petri dish per line) sown each time, and effective germination was defined as the formation of 3 mm or longer roots. Germination was calculated as the mean of three replications. Using Student's t-test, asterisks indicate significant differences: non-significant (ns). Data are shown as mean ± SD from three independent experiments.

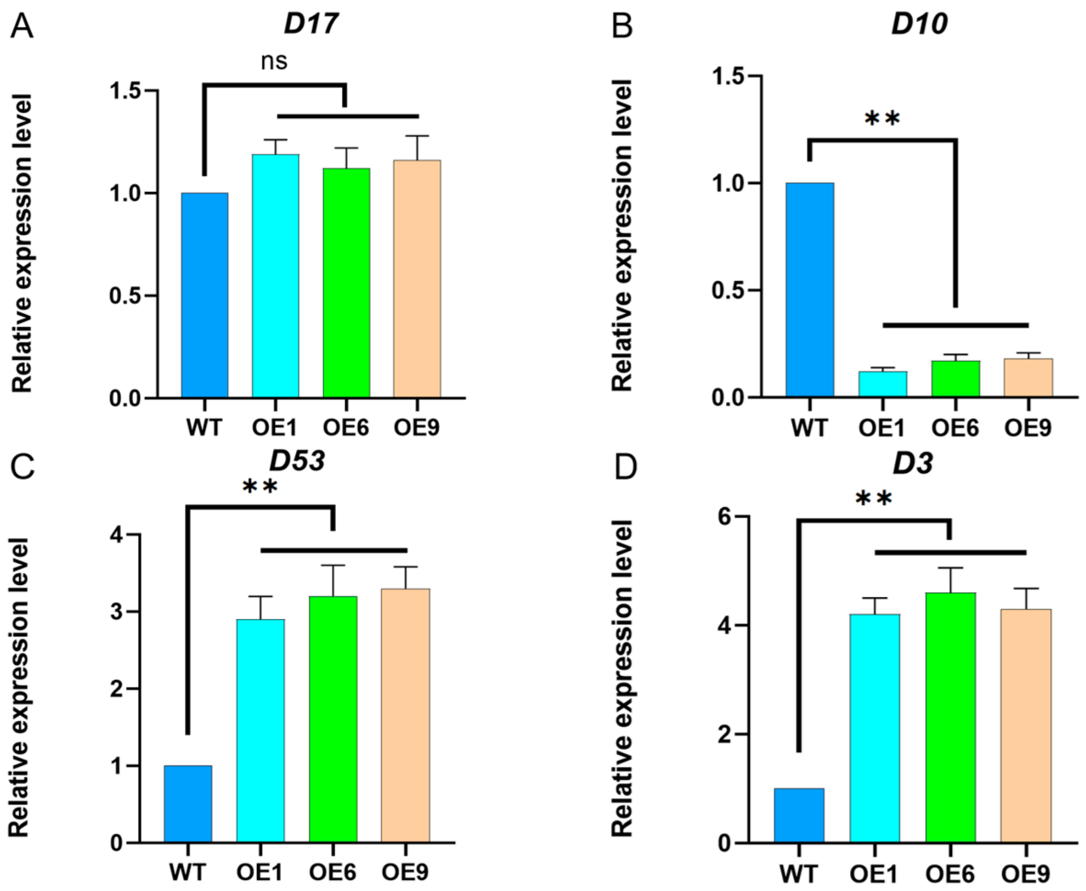

Figure S3. Analysis of the expression of key genes in strigolactones biosynthesis and signal transduction. (A, B) expression analysis of key genes *D17* and *D10* in strigolactones synthesis pathway; (C, D) expression analysis of key genes *D53* and *D3* in strigolactones signaling pathway; The expression levels of WT lines were normalized. Using Student' s t-test, asterisks indicate significant differences: non-significant (ns) and $p < 0.01$ (**). Data are shown as mean ± SD from three independent experiments.

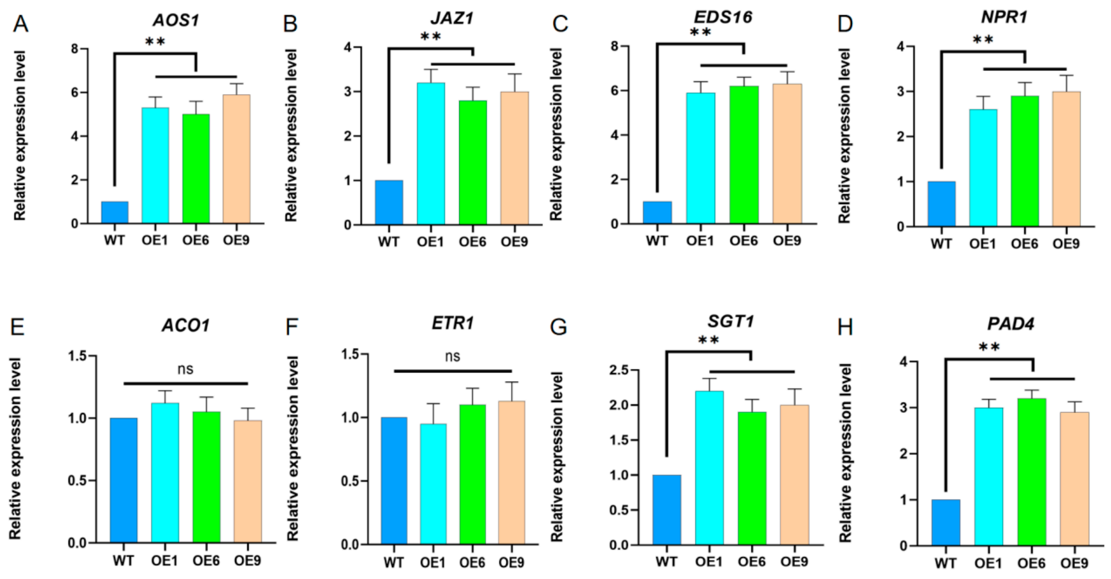

Figure S4. Three days after inoculation with the pathogen *Pst. DC3000* (*Pseudomonas syringae pv. tomato DC3000*), the expression profiles of key genes involved in the biosynthesis and signalling processes of SA, JA and ET, as well as of two *R*-gene-associated genes, were reassessed. (A, B) expression of key genes in jasmonic acid (JA) synthesis and signaling pathways; (C, D) expression of key genes in salicylic acid (SA) acid synthesis and signaling pathways; (E, F) expression of key genes in ethylene (ET) synthesis and signaling pathways; (G, H) *R* gene related key gene expression detection; The expression levels of WT lines were normalized. The experiment was repeated three times, each time using eight *Arabidopsis* (two each of WT lines, OE1 lines, OE6 lines, and OE9 lines). At 3 dpi, all rosette leaves of each *Arabidopsis* were taken for RNA extraction and quantitative fluorescence detection. The experiment repeated three times. Using Student's t-test, asterisks indicate significant differences: non-significant (ns) and $p < 0.01$ (**). Data are shown as mean ± SD from three independent experiments.
